# Brain death-like changes: A case report of anti-GQ1b antibody syndrome

**DOI:** 10.1097/MD.0000000000032749

**Published:** 2023-02-17

**Authors:** Jia Tian, Yi Zhou, Hemei Cui, Limiao Zhang, Yan Xue, Lidou Liu

**Affiliations:** a Neurocritical Care Unit, Department of Neurology, The Second Hospital of Hebei Medical University, Shijiazhuang, Hebei, China.

**Keywords:** anti-GQ1b antibody syndrome, BBE, GBS, MFS

## Abstract

Anti-GQ1b antibody syndrome originates in the peripheral nervous system or the central nervous system. Various overlapping syndromes and atypical manifestations are also seen in clinic. It is rare to see multiple sites (almost all targets) of central and peripheral damage at the same time, resulting in brain death-like changes in patient.

**Patient concerns:**

This is a 55-year-old female patient, with a history of prodromal upper respiratory tract infection, began with headache, quickly developed body paralysis, eye paralysis, disturbance of consciousness, apnea, and tested positive for anti-GQ1b antibody. The patient is diagnosed clearly, the disease progresses rapidly, and almost all GQ1b sites in the central nervous system and peripheral nervous system are involved, which is rare.

**Diagnoses:**

Anti-GQ1b antibody syndrome.

**Interventions and outcomes:**

The patient was treated with tracheal intubation, ventilator assisted breathing, and immunoglobulin. The patient recovered quickly and was discharged after about 30 days in hospital.

**Lessons:**

The concept of anti-GQ1b antibody syndrome is not only beneficial for clinical diagnosis, but also beneficial for understanding the continuous disease spectrum with the same etiology and different clinical manifestations. The pathogenesis of each subtype has not been fully defined. There are mild patients with isolated syndromes and severe patients with multiple subtypes overlapping. Encounter severe patients but also active response, the general prognosis is good.

## 1. Introduction

Anti-GQ1b antibodies were initially thought to be more common in Miller Fisher syndrome (MFS), and with the gradual expansion of the detection range, more and more clinical cases have been included in this spectrum of disease. In 2001, anti-GQ1b antibody syndrome was first proposed by Odaka,^[1]^ to summarize the spectrum of diseases with the same mechanism but different clinical manifestations.^[2,3]^ It originates in the peripheral nervous system (PNS) or the central nervous system (CNS).^[3]^ A variety of phenotypes are known in the origin of PNS, mainly manifested as ophthalmic paralysis MFS, limb weakness (Guillain-Barré syndrome with ophthalmic paralysis) or sensory ataxia (acute sensory ataxic neuropathy). In contrast, in CNS origin, only 1 phenotype is known, Bickerstaff brainstem encephalitis. It accounts for the main proportion of brain stem encephalopathy. Clinically, there are 3 kinds of symptoms: ocular paralysis, ataxia and consciousness disorder.^[4]^ Various overlapping syndromes and atypical manifestations are also seen in clinic,^[4,5]^ however, it is rare to see multiple sites (almost all targets) of central and peripheral damage at the same time, resulting in brain death-like changes in patient. We present a case with rapidly progressive quadriplegia, respiratory depression, altered consciousness, and multiple central and peripheral target involvement, presenting with clinical changes similar to brain death.

## 2. Case report

**Figure 1.**
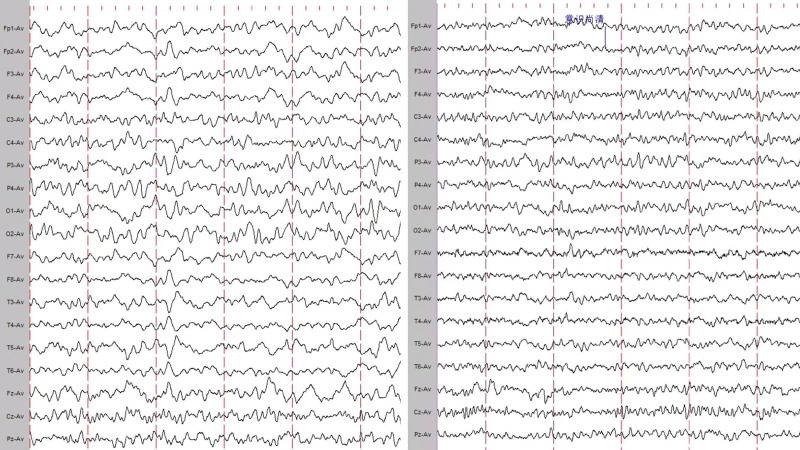
Results of the patient’s 19-lead EEG: (A) day 10 EEG and (B) day 21 EEG. EEG = electroencephalogram.

**Figure 2.**
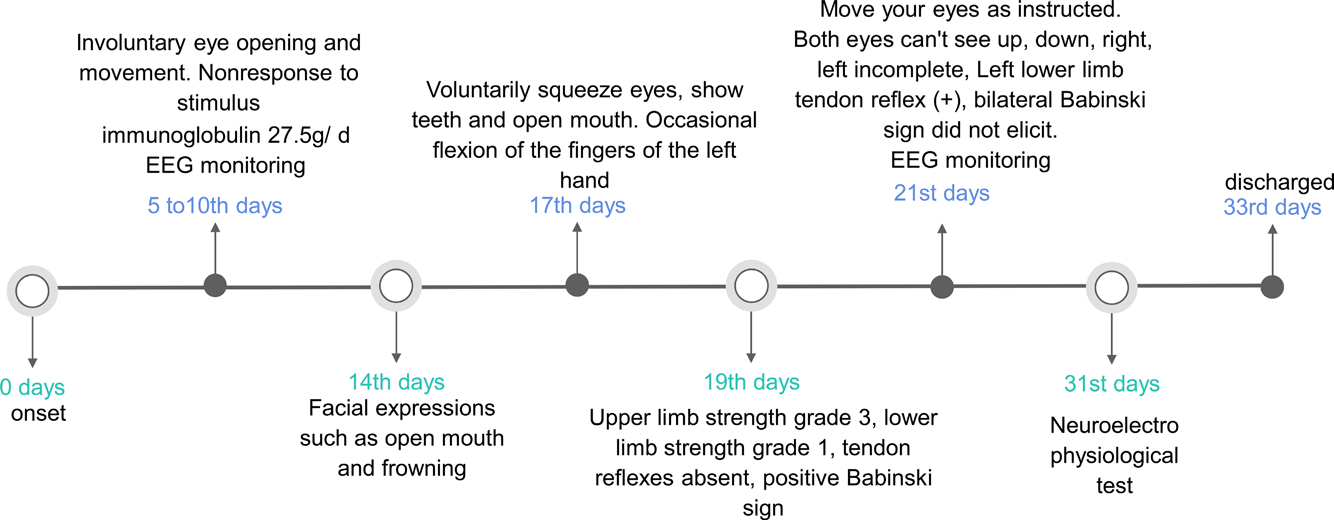
The overall prognosis, auxiliary examination and medication.

A 55-year-old female patient was admitted to the hospital mainly due to intermittent headache for 2 days, progressive enhancement of limb weakness for 1 day, and unconsciousness for 4 hours. The patient developed a headache after dinner 2 days ago, presenting with right temporo-occipital pain and tightness. One day ago, numbness and weakness appeared on the right limb, and she could stand and walk with difficulty. Later, weakness appeared on the left limb, and she could not stand or walk. Accompanied by the right and left eyelid lifting weakness, weakness of chewing, slurred speech. This is followed by increased sleep, apnea, and difficulty urinating. Presented to our hospital with unconsciousness 4 hours ago. Admitted to hospital for physical examination: shallow coma, double pupils are round and equal in diameter, about 2.5 mm, dull light reflex, uncooperative eye movement in all directions, no nystagmus. Bilateral frontal striae symmetrical, no nasolabial sulcus light. Muscle strength examination of the extremities was not cooperative, strong tingling showed contraction of the limbs, muscle tone of the limbs was decreased, tendon reflex of the limbs disappeared, bilateral Babinski sign was positive, sensory and ataxia movement examination was not cooperative, cervical resistance (-). After admission, she was given endotracheal intubation and ventilator assisted breathing due to type I respiratory failure. Lumbar puncture was performed, and the results were shown in Table 1. MRI + DWI + MRA + CEMRV + enhancement: No obvious signal of limited diffusion was observed on DWI; MRA: part of cerebellar artery was not shown; There was no obvious abnormality in craniocerebral vein CEMRV. No abnormality was observed with direct craniocerebral magnetic enhancement. Cerebrospinal fluid DNA sequencing and RNA sequencing: no abnormal cerebrospinal fluid and blood were found. Antibodies associated with autoimmune encephalitis: negative. Myasthenia gravis antibody profile test (serum): negative. Ganglioside antibody spectrum was used to detect anti-GQ1b IgG (+) and anti-GT1A IgG (+). Neuroelectrophysiological test: Motor fibers of left common peroneal nerve and right median nerve were damaged; Sensory fibers of double median nerve, double ulnar nerve and double superficial peroneal nerve were damaged; The incubation period of “F” wave of right median nerve and double ulnar nerve is still in the normal range, with the occurrence rate of 20% to 30%↓; The latent period, amplitude and conduction velocity of nerves in both upper and lower limbs of the rest tested were in the normal range. electroencephalogram (EEG) (day 10): mild to moderate abnormalities; Full conductivity diffuse mixed slow wave emission. EEG (day 21): mild to moderate abnormalities; Full conductivity diffuse mixed slow wave emission. Better than before. See Figure 1. The patient was given immunoglobulin 0.4g/kg/day from the 5th day of onset for 5 consecutive days. Fourteen days after the onset of the disease, the patient’s condition showed a trend of improvement and facial expression gradually appeared. On 19 days after the onset of the disease, the muscle strength of the limbs began to recover, and finally the consciousness was completely recovered. On the 33rd day of the disease, the patient was discharged from the hospital. At the time of discharge, the patient was alert, the eyeballs could move in all directions, there was no facial paralysis, the tongue was not skewed, the muscle strength of the limbs was grade 4, the muscle tension of the upper limbs was slightly reduced, the muscle tension of the lower limbs was normal, and the tendon reflex of the lower limbs (++) bilateral Babinski sign was negative. Figure 2 showed the overall prognosis, auxiliary examination and medication.

**Table 1:** The results of Lumbar puncture.

Items	3 days after onset	10 days after onset	15 days after onset	21 days after onset
Pressure (mmH_2_O)	180	260	270	200
Total cell (×10^6^/L)	2108	495	52	15
WBC (×10^6^/L)	8	9	12	3
CL (mmol/L)	122.3	118.6	122.6	125.3
Protein (g/L)	0.57	0.49	0.72	0.85
Glucose (mmol/L)	6.29	3.98	5.68	2.82

WBC = white blood cell.

## 3. Discussion

This middle aged female patient, with a history of prodromal upper respiratory tract infection, began with headache, quickly developed body paralysis, eye paralysis, disturbance of consciousness, apnea, and tested positive for anti-GQ1b antibody. The patient is diagnosed clearly, the disease progresses rapidly, and almost all GQ1b sites in the CNS and PNS are involved, which is rare.

Anti-GQ1b antibodies cause reversible damage to the brain stem.^[3,6]^ One of the largest series of reports published showed,^[7]^ T2-weighted images 11% of 47 Bickerstaff brainstem encephalitis patients had high intensity abnormalities in the pons (n = 4), thalamus (n = 2), cerebellum (n = 1), medulla oblongata (n = 1), midbrain (n = 1), superior cerebellar peduncle (n = 1), or corpus callosum (n = 1). Boeun Lee et al^[5]^ conducted a retrospective analysis of 16 high-resolution MR examinations in 15 patients with anti-GQ1b antibody syndrome, as 1 patient (case 5) had repeated MRI examinations for delayed facial paralysis. Median time from onset to MR Was 3 days (range 0–21 days). All MR examinations showed no abnormal brain stem. Previous studies have also reported that MR Brain imaging results do not always correlate with clinical symptoms.^[8,9]^ MRI scan and enhancement were also performed on our patient, and no abnormalities were found. But we still think the patient has CNS damage. First, the patient’s physical examination was positive for bilateral Babinski sign, indicating the presence of pyramidal tract injury. Second, the patient’s EEG is abnormal, suggesting brain dysfunction; Third, after the patient complied with the instructions, we asked whether the patient had any memory of the previous hospitalization, but the patient could not recall, indicating that the patient did have consciousness disorder in the early stage (in order to evaluate the patient’s consciousness, we stopped drugs affecting consciousness early). As for the negative MRI of the patient, it was considered that the patient’s damage was incomplete and did not cause structural damage, so the MRI was not developed, and the patient recovered quickly in the later stage, which also supported our idea. With positive bilateral pyramidal tract sign combined with disturbance of consciousness, the localization could be bilateral cerebral hemispheres and bilateral thalamus and brainstem. Combined with negative MRI of the patient, considering the location still in the brain stem is more likely, which is also consistent with the GQ1b distribution location.

Anti-GQ1b antibodies can cause damage to peripheral nerves, including cranial and spinal nerves. Our patient’s earliest judgment of consciousness recovery was that he could open his mouth as instructed. At that time, the eyeballs moved in all directions and the pupil’s reflex to light was limited, and the face was expressionless. Then gradually appeared sensitive to light reflex recovery, facial simple expression, eye movement also gradually recovered. These symptoms can be localized in the brain stem or in the corresponding cranial nerves. We do not rule out that the brain stem was damaged in corresponding positions, but there were multiple brain stem nerve nuclei damaged at the same time, and there was no positive change in the MRI, which is difficult to explain. Therefore, we speculate that these symptoms of the patient can not be ruled out as the manifestation of the corresponding cranial nerve damage. Since we have not conducted cranial nerve nuclear magnetic and related electrophysiological examination, we do not have direct evidence to prove the existence of cranial nerve injury. However, multiple literatures support that anti-GQ1b antibodies can cause damage to multiple cranial nerves, and studies have shown that the median cranial nerve involved in patients is 4, ranging from 0 to 8. The most commonly involved cranial nerve was the third (88%), followed by the 6th (56%), 7th (25%), fifth (6%) and 8th (6%) nerves.^[5,7,10–12]^ It is consistent with our patient’s symptoms. GQ1b is mainly expressed in the paranoid regions of these extramedullary parts of the cranial nerves.^[13]^

As the patient’s muscle strength was close to normal during the peripheral nerve electrophysiological detection, and the clinical symptoms were obviously recovered, the monitoring results showed that the patient’s peripheral nerve injury was not obvious, and there was no clear evidence of localization. However, the literature suggested that the possible injury pathology showed that it was the dysfunction of the axon membrane junctions and nodes and paranodes leading to nerve conduction failure and block, without myelin loss and regeneration. A dynamic nerve conduction study of 15 patients with MFS revealed reversible nerve block similar to AMAN in 6 patients without evidence of demyelination and regeneration. Our patient had a rapid recovery of symptoms and was also consistent with reversible block, and the electrophysiology had recovered at the time of our examination. Of course, patients with positive Babinski sign cannot exclude body paralysis due to central causes.^[14]^

Since the intrafusal fiber are rich in gangliosides – GQ1b and GD1b, the molecular simulation of anti-GQ1b, GD1b and GQ1b/GD1b complex antibodies bind to the corresponding gangliosides, resulting in the failure of the action potential formation of I a afferent nerve fibers, resulting in the loss of tendon reflex and sensory ataxia. In our patient, tendon reflexes disappeared in the early stage, and due to the patient’s consciousness disorder in the early stage, it is difficult to conduct the examination of the ataxia movement.

The reason for the difference in clinical presentation among patients with anti-GQ1b antibody syndrome is unclear. Some researchers have hypothesized that because gangliosides are expressed differently in different parts of the nervous system, differences in specificity of anti-GQ1b antibodies are related to various clinical manifestations.^[2,15]^ Individual differences in GQ1b expression sites and the availability of anti-GQ1b antibodies may be the cause of different clinical symptoms. The cross-reactivity of anti-GQ1b antibodies with other ganglioside antibodies varies depending on clinical symptoms and relevant anatomical site.^[2,15]^

## 4. Conclusion

The concept of anti-GQ1b antibody syndrome is not only beneficial for clinical diagnosis, but also beneficial for understanding the continuous disease spectrum with the same etiology and different clinical manifestations. The pathogenesis of each subtype has not been fully defined. There are mild patients with isolated syndromes and severe patients with multiple subtypes overlapping. Encounter severe patients but also active response, the general prognosis is good.

## Author contributions

**Conceptualization:** Jia Tian.

**Data curation:** Jia Tian.

**Formal analysis:** Yi Zhou

**Investigation:** Yi Zhou.

**Project administration:** Lidou Liu.

**Resources:** Hemei Cui.

**Software:** Hemei Cui.

**Supervision:** Limiao Zhang.

**Validation:** Limiao Zhang, Yan Xue.

**Visualization:** Yan Xue.

**Writing – original draft:** Jia Tian, Lidou Liu.

**Writing – review & editing:** Lidou Liu.
